# Issues around vulnerability among people attended by a Portuguese community-based association: a qualitative secondary analysis

**DOI:** 10.1192/j.eurpsy.2024.1706

**Published:** 2024-08-27

**Authors:** C. Laranjeira, A. I. Querido

**Affiliations:** ^1^ School of Health Sciences; ^2^ciTechCare, Polytechnic University of Leiria, Leiria, Portugal

## Abstract

**Introduction:**

Despite vulnerability being a poorly understood concept is a key concept in health and social care disparities. Typically, vulnerable groups include individuals with physical and/or mental disabilities, children, the elderly, members of the lower social classes, and refugees. In Portugal, the pandemic was responsible for worsening inequalities in access to health and social care for the most vulnerable. To the best of our knowledge, there is a dearth of qualitative research on vulnerability from the viewpoint of those who are vulnerable or work with the most vulnerable.

**Objectives:**

As expressions of vulnerability are strongly influenced by cultural factors, this study aims to examine issues of vulnerability among people who attend and work in a Portuguese community-based association.

**Methods:**

Secondary analysis of qualitative data from twelve vulnerable people and fifteen professionals who attended these people. The manifestations of the vulnerability reported by participants included being homeless, being a migrant, having an infectious disease, being drug dependent, living with socioeconomic difficulties (unemployment), and experiencing a process of loss and grief. They also reported having a mental or physical health problem, or both. Depression and anxiety were the most often reported mental health disorders. Regarding the academic background of professionals, most of them (n = 12) are from social sciences (e.g., social workers, social mediators, and psychologists).

**Results:**

Three main themes emerged from the study: (1) meanings of human vulnerability; (2) barriers to vulnerability mitigation; and (3) approaches to addressing vulnerability. Our findings revealed that vulnerability is a very dynamic process of openness to conditions that impact individual outcomes. However, there is a conceptual gap: being vulnerable is perceived as something negative, but vulnerability also has the potential to change priorities in life for the better. Some participants emphasized the importance of self-care to avoid becoming vulnerable themselves, particularly in terms of mental health.

**Conclusions:**

Understanding the social determinants of vulnerability is necessary to achieve satisfactory care for human groups. Practitioners need to be aware of these larger societal dynamics, understand them, and make sure their services are responsive to cultural differences. In order to develop interventions that promote social and health outcomes, practitioners should be encouraged to share knowledge on best practices.

**Disclosure of Interest:**

None Declared

